# Hindlimb muscle representations in mouse motor cortex defined by viral tracing

**DOI:** 10.3389/fnana.2023.965318

**Published:** 2023-05-25

**Authors:** Lauren Maurer, Maia Brown, Tamandeep Saggi, Alexia Cardiges, Christi L. Kolarcik

**Affiliations:** ^1^Department of Pathology, University of Pittsburgh School of Medicine, Pittsburgh, PA, United States; ^2^Department of Bioengineering, University of Pittsburgh, Pittsburgh, PA, United States; ^3^LiveLikeLou Center for ALS Research, University of Pittsburgh Brain Institute, Pittsburgh, PA, United States; ^4^McGowan Institute for Regenerative Medicine, University of Pittsburgh, Pittsburgh, PA, United States; ^5^Center for the Neural Basis of Cognition, University of Pittsburgh, Pittsburgh, PA, United States

**Keywords:** cortical representations, motor cortex, hindlimb muscle, motor control, neural circuit

## Abstract

**Introduction:**

Descending pathways from the cortex to the spinal cord are involved in the control of natural movement. Although mice are widely used to study the neurobiology of movement and as models of neurodegenerative disease, an understanding of motor cortical organization is lacking, particularly for hindlimb muscles.

**Methods:**

In this study, we used the retrograde transneuronal transport of rabies virus to compare the organization of descending cortical projections to fast- and slow-twitch hindlimb muscles surrounding the ankle joint in mice.

**Results:**

Although the initial stage of virus transport from the soleus muscle (predominantly slow-twitch) appeared to be more rapid than that associated with the tibialis anterior muscle (predominantly fast-twitch), the rate of further transport of virus to cortical projection neurons in layer V was equivalent for the two injected muscles. After appropriate survival times, dense concentrations of layer V projection neurons were identified in three cortical areas: the primary motor cortex (M1), secondary motor cortex (M2), and primary somatosensory cortex (S1).

**Discussion:**

The origin of the cortical projections to each of the two injected muscles overlapped almost entirely within these cortical areas. This organization suggests that cortical projection neurons maintain a high degree of specificity; that is, even when cortical projection neurons are closely located, each neuron could have a distinct functional role (controlling fast- versus slow-twitch and/or extensor versus flexor muscles). Our results represent an important addition to the understanding of the mouse motor system and lay the foundation for future studies investigating the mechanisms underlying motor system dysfunction and degeneration in diseases such as amyotrophic lateral sclerosis and spinal muscular atrophy.

## 1. Introduction

Descending motor pathways from the cerebral cortex to the spinal cord mediate the movement of muscles throughout the body for behaviors ranging from basic locomotion to dexterous object manipulation. In rodents, these cortical projections originate in layer V and connect to brainstem nuclei and/or spinal cord interneurons. In humans and some non-human primates, cortical projections originating in layer V can directly impact the activity of motor neurons, and prior work indicates that these direct connections mediate highly-skilled motor behaviors in these species (Lemon, 2008; Rathelot and Strick, 2009; Strick et al., 2021). Together, these direct and indirect pathways from the cerebral cortex determine how and when voluntary movement occurs. An understanding of these pathways is critical to identifying and targeting treatments for dysfunction associated with pathological conditions that affect the motor system.

Although the mouse is widely used to study the neurobiology of disease, the organization of the mouse motor system has not yet been fully delineated. Previous work by others has investigated the neural circuitry underlying forelimb muscles in mice, revealing a selective synaptic connectivity matrix between the cortex, brainstem, and spinal motor neurons that regulates forelimb movement (Esposito et al., 2014; Ueno et al., 2018). However, the areas of the cortex and brainstem that are directly responsible for controlling the spinal cord motor neurons that innervate hindlimb muscles have not been fully characterized.

Another important consideration in understanding descending control over movement is the presence of different motor unit types. Muscles are composed of varying types of muscle fibers including fast fatigue-resistant, fast fatiguable, and slow-twitch, and the motor neurons that innervate each muscle fiber type have different anatomical, physiological, and functional properties. Henneman’s size principle has established that small motor units and small motor neurons are recruited before large motor units and large motor neurons (Henneman et al., 1965). These small motor units tend to be slow-twitch units that are recruited prior to fast fatigue-resistant and fast fatigable units. Differential loss of muscle fibers based on type has been observed in conditions ranging from muscular dystrophies to cancer cachexia [reviewed in (Ciciliot et al., 2013)]. A preferential loss of fast-twitch muscle fibers has also been reported in rodent models of amyotrophic lateral sclerosis (ALS) (Frey et al., 2000; Pun et al., 2006; Hegedus et al., 2007) despite it being regarded as a disease of the motor neuron. A better understanding of differences in the cortical innervation of specific motor unit types is essential for addressing the neural mechanisms underlying these complex conditions.

In this study, we used trans-synaptic virus-based tracing to identify the origin of cortical control of motor neurons that innervate distinct hindlimb muscles in the mouse. We chose rabies virus to interrogate the motor circuitry because it is transported exclusively in the retrograde direction and trans-synaptically in a time-dependent manner (Ugolini, 2010); through the adjustment of survival time, retrograde transneuronal transport of rabies virus enables identification of multiple nodes in a chain of synaptically-linked neurons (Kelly and Strick, 2003). We investigated the innervation of two muscles with either predominantly slow-twitch muscle fibers (soleus, 50% type I) or predominantly fast-twitch muscle fibers [tibialis anterior (TA), 90% type II] (Frey et al., 2000). We show that the motor neurons of these two hindlimb muscles were infected by retrograde transport of rabies virus at different rates. Labeling of the soleus motor neuron pool occurred before that of the TA motor neuron pool, indicating differential uptake of rabies virus at the level of muscle. However, following viral uptake into the nervous system at the neuromuscular junction, the rate of retrograde transneuronal transport was not significantly different for soleus versus TA, and similar anatomical nodes in the spinal cord and brainstem were involved in mediating virus transport from both TA and soleus to layer V neurons in the cerebral cortex. Further, density-based analyses revealed that the cortical regions associated with the control of each hindlimb muscle exhibited considerable overlap. These results suggest that cortical neurons retain some differential specificity related to the muscle fiber types innervated by their target motor neurons.

## 2. Materials and methods

### 2.1. Surgical procedures

All surgical procedures were performed in accordance with those outlined by the Association for Assessment and Accreditation of Laboratory Animal Care (AAALAC) and the National Institutes of Health Guide for the Care and Use of Laboratory Animals. The experimental protocol was approved by the Institutional Animal Care and Use Committee as well as the Biosafety Committee of the University of Pittsburgh. Biosafety practices followed those outlined in Biosafety in Microbiological and Biomedical Laboratories (Department of Health and Human Services Publication No. 93-8395). Procedural details regarding the handling of virus and virus-infected animals have been outlined previously (Kelly and Strick, 2000). Animals were housed in the facilities of the University of Pittsburgh, Division of Laboratory Animal Resources, and all efforts were made to minimize the number of animals used as well as their suffering.

All procedures were performed using aseptic techniques and under anesthesia. Adult male mice on the C57Bl/6 background (The Jackson Laboratory, Farmington, CT) weighing approximately 30 g were given buprenorphine (0.1 mg/kg) prior to and following surgery. Animals were anesthetized with 2.5% isoflurane in oxygen at approximately 150 mL/min and monitored closely throughout the procedure by observing changes in respiratory and heart rates. Animal temperature was maintained throughout the procedure using a heating pad, and ophthalmic ointment was applied to the eyes while under anesthesia.

Once anesthetized, animals were placed onto their left side and the right hindlimb shaved and then cleaned with betadine. The right hindlimb was draped with a sterile towel, and a longitudinal incision from approximately the knee to the ankle was made to expose either the TA or the soleus muscle. Rabies virus (CVS-N2c; 1 × 10^9^ pfu/mL; supplied by M. Schnell, Thomas Jefferson University, Philadelphia, PA) was injected using a 30-gauge Hamilton syringe along the length of the muscle at increasing volumes (10, 19, and 40 μL) for TA muscle in initial experiments and then at a volume proportional to the weight of each muscle for subsequent experiments [approximately 3 μL for soleus and approximately 19 μL for TA based on muscle weights of 6.58 ± 2.09 mg and 42.17 ± 6.33 mg, respectively (Charles et al., 2016)]. Only one of these two muscles (either soleus or TA) was injected in each animal. Once injections were completed, sterile saline was used to flush the area surrounding the injection site. The skin was sutured, and the animals were returned to cages designed for the housing of virus-infected animals. Animals were singly-housed and given free access to food and water following surgery with environmental enrichment provided in the isolation suite throughout the survival period. Animals displayed no symptoms of rabies virus infection over the survival times utilized in this study. Current evidence suggests that rabies virus is transported transneuronally in all types of systems and across all types of synapses (Kelly and Strick, 2000, 2003; Hoshi et al., 2005; Ugolini, 2010).

### 2.2. Survival period

The survival times varied from approximately 24 h to approximately 93 h. The total number of animals used in this study was 96 (*n* = 44 for soleus muscle injections; *n* = 52 for TA muscle injections; *n* = 4 animals/time point). For soleus muscle injections, one animal sacrificed at the 24-h survival time did not have labeling and four animals at the 93-h survival time had labeling beyond 4th order and were not used in the analyses. For TA muscle injections, one animal sacrificed at the 29-h survival time and one animal sacrificed at the 36-h survival time did not have labeling and one animal at the 75-h survival time could not be evaluated because of damage during sectioning and were not used in the analyses. Therefore, the analyses presented here includes data from 88 total animals (*n* = 39 for soleus muscle injections; *n* = 49 for TA muscle injections). At the designated survival period, animals were deeply anesthetized with sodium pentobarbital (100 mg/kg) before being transcardially perfused first with 0.1 M phosphate buffer followed by either 10% phosphate buffered formalin or 4% paraformaldehyde. Following perfusion, the brain and spinal cord were removed and placed in fixative at 4*^o^*C for up to three days and then transferred to phospho-tris-azide. Brains were embedded in gelatin and serial coronal sections (50 μm) of the cerebral cortex and cerebellum were collected. Lumbar spinal cord blocks (from approximately T13 to S2) were embedded in gelatin and cross-sections (50 μm) were collected.

### 2.3. Histological procedures

Every 10th section of the brain and spinal cord was stained with cresyl violet to enable analysis of cytoarchitecture. Even-numbered sections of the brain and spinal cord were used to evaluate the presence of rabies-positive neurons. Immunohistochemical reactions on free-floating brain and spinal cord sections using the avidin-biotin peroxidase method (Vectastain, Vector Laboratories, Burlingame, CA) were used to identify rabies-infected neurons. These reactions utilized a mouse monoclonal antibody (31G10, used at a concentration of 0.1 μg/mL, supplied by M. Schnell, Thomas Jefferson University, Philadelphia, PA) isolated previously (Raux et al., 1997) and used successfully in multiple tracing studies (Ruigrok et al., 2008, 2016; Salin et al., 2008; Coulon et al., 2011; Suzuki et al., 2012; Kasumacic et al., 2015). Following overnight incubation at 4*^o^*C, sections were washed in buffer and biotinylated secondary antibody was added at a dilution of 1:500. The signal was then amplified using ABC Reagent with 3,3’-diaminobenzidine (DAB) as the chromagen. Reacted sections were mounted on gelatin-covered glass slides, air-dried, washed, and coverslipped with Cytoseal.

For immunofluorescence, free-floating tissue sections were blocked in normal donkey serum in phosphate buffered saline (PBS). Primary antibodies [the mouse monoclonal antibody described above (31G10) and choline acetyltransferase (ChAT; goat polyclonal; ProSci, Poway, CA), a marker of cholinergic neurons] were added for 48 h at 4*^o^*C, and the appropriate fluorescence-conjugated secondary antibodies were added for 1–2 h at room temperature at a dilution of 1:500. Hoechst 33342 (Invitrogen) was used as a nuclear counterstain according to the manufacturer’s instructions. Sections were mounted with SlowFade Gold (Invitrogen). Digital images were acquired with a fluorescence microscope (FluoView 1000, Olympus, Inc., Tokyo, Japan). Detection parameters were optimized for each fluorophore and consistent settings used for all images with image contrast and brightness adjusted in Adobe Photoshop (Adobe Systems Inc., San Jose, CA).

### 2.4. Neuronal quantification

Profile counts were obtained by counting every cell in every other section of the entire region of interest (i.e., from T13 to S2 for the lumbar spinal cord and for the entire brain for the cortex). Rabies virus is an RNA virus that remains confined to the cytoplasm and does not enter the nucleus. Cell profiles without an apparent nucleus and lacking an apical dendrite are more likely to be partial cells and were not counted. Spinal motor neurons were positively identified by labeling with rabies virus (31G10 antibody) in every other section of the lumbar spinal cord. Counts of spinal motor neurons were performed in animals in which no spinal interneurons were labeled with rabies virus; these counts were doubled to account for the sections that were not evaluated (Table 1). Cortical neurons in layer V positive for rabies virus were quantified from every other coronal section of the brain. Counts of neurons labeled in cortical layer V were performed in animals in which cortical labeling was confined to layer V. The same assessment was done for each hindlimb muscle at several survival times (Table 2). For all assessments, the entire region of interest was sectioned at an interval of two with every other section being sampled, and, for each section, the entire section was sampled for positive cells. All assessments were performed in a blinded fashion.

**TABLE 1:** Quantification of spinal motor neuron labeling in animals with labeling restricted to first-order motor neurons only following rabies virus (1 × 10^9^ pfu) injection.

Survival time (*n* = number of animals)	Spinal motor neurons (average ± SD)
**Tibialis anterior**	
29 h (*n* = 3)	4.7 ± 2.3
36 h (*n* = 3)	14.0 ± 5.3
40 h (*n* = 4)	18.0 ± 11.7
44 h (*n* = 3)	10.0 ± 7.2
**Soleus**	
24 h (*n* = 3)	16.0 ± 10.4
30 h (*n* = 4)	23.0 ± 4.8
36 h (*n* = 4)	22.6 ± 5.7

Counts have been doubled to account for the sampling of every other spinal cord section.

**TABLE 2:** Quantification of layer V cortical neuron labeling in the hemisphere contralateral to the injection site in animals with labeling restricted to third-order layer V neurons only following rabies virus (1 × 10^9^ pfu) injection.

Survival time (*n* = number of animals)	Layer V neurons (average ± SD)
**Tibialis anterior**	
58 h (*n* = 1)	2.0 ± 0.0 (not included in density analyses)
60 h (*n* = 1)	1.0 ± 0.0 (not included in density analyses)
63 h (*n* = 1)	6.0 ± 0.0
69 h (*n* = 4)	9.0 ± 8.5
75 h (*n* = 2)	86.5 ± 16.26
84 h (*n* = 4)	309.5 ± 251.84
**Soleus**	
54 h (*n* = 2)	1.5 ± 0.71 (not included in density analyses)
60 h (*n* = 4)	4.75 ± 0.50
67 h (*n* = 4)	63.0 ± 29.45

### 2.5. Cortical mapping

Brain and spinal cord sections were examined using bright-field microscopy and polarized light illumination. To create cortical maps, a computer-based charting system was used to delineate section outlines, rabies-labeled neurons, gray-white matter boundaries, and other neuroanatomical features (i.e., corpus callosum, M1/S1 border, M1/M2 border, rhinal fissure) from every other section of the mouse brain as described previously (Dum and Strick, 1991). The nomenclature and boundaries for cortical areas were based on a standard atlas of the mouse brain (Paxinos and Franklin, 2019). Custom laboratory software enabled the interactive aligning of charts of these individual sections. An “unfolding” line was then drawn along the top of cortical layer V and labeled neurons were projected to that line. The entire sequence of unfolded lines was then combined to generate high-resolution unfolded cortical maps as described previously (Dum and Strick, 1991). These maps, developed for both the TA and soleus muscles, display the location of labeled neurons in a two-dimensional representation of the cerebral cortex. Reconstructed cortical maps from experimental animals were aligned and scaled to a standardized atlas using common features such as the locations of the midline and the agranular-granular border between M1 and S1 as described previously in rats (Levinthal and Strick, 2012). Density-based analyses were performed to assess areas with peak labeling after peripheral muscle injections. To determine the density of labeling, we counted infected neurons in successive 100 μm by 100 μm bins.

### 2.6. Statistical analyses

Statistical analyses were performed using Graph Pad Prism 9 (Graph Pad Software, San Diego, CA). All data are presented as mean ± SD.

## 3. Results

**FIGURE 1:**
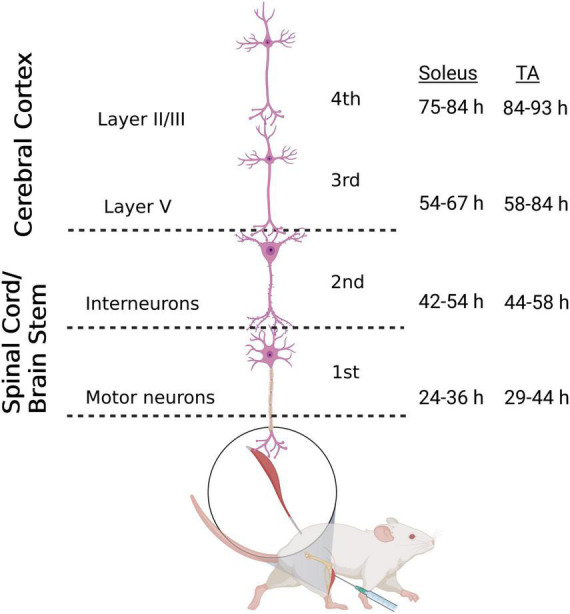
Retrograde transneuronal transport of rabies virus through the neural circuits that innervate muscles of the hindlimb. Following intramuscular injection, rabies virus is transported through a chain of synaptically-connected neurons. Once injected into a peripheral muscle [i.e., tibialis anterior (TA) or soleus], retrograde transport of the virus results in infection of motor neurons in the ventral horn of the spinal cord (1st order) then interneurons in the intermediate zone of the spinal cord and/or in the brainstem (2nd order) and then neurons in layer V of the cerebral cortex (3rd order) in a time-dependent manner. From layer V of the cerebral cortex, layer II/III neurons of the cerebral cortex, as well as other areas projecting to layer V, are infected (4th order). The observed time course of this retrograde labeling at each location is summarized for soleus and TA. Each stage of transport in the figure is numbered as described in the text. Figure created with BioRender.com.

The N2c strain of rabies virus is transported trans-synaptically in the retrograde direction via motor efferent pathways following injection into peripheral muscles (Rathelot and Strick, 2006). We used this approach to identify the anatomical nodes within the brain and spinal cord with the most direct synaptic connections to the motor neurons responsible for innervating hindlimb muscles in the mouse. Following unilateral virus injections into either the tibialis anterior (TA) or soleus muscle, multiple stages of viral replication and transneuronal transport resulted in the sequential infection of synaptically interconnected neurons (Figure 1). Through careful adjustment of survival time (24–93 h), rabies-infected cells were first observed in spinal motor neurons and, subsequently, in spinal interneurons and brainstem nuclei prior to transport to cortical layer V and then cortical layers II/III and VI. Animals did not display symptoms during the extended survival periods used in these experiments, consistent with previous reports using rabies virus in the rodent (Levinthal and Strick, 2012).

### 3.1. Efficiency of spinal motor neuron and cortical layer V transduction

**FIGURE 2:**
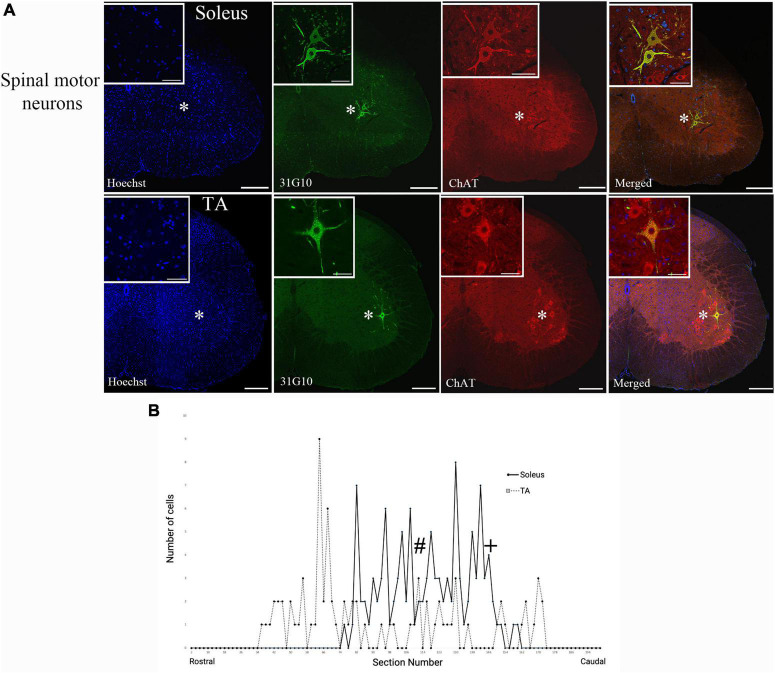
Ventral horn location and spinal cord distribution of first-order motor neuron labeling. **(A)** Spinal motor neurons labeled following soleus muscle injections were located more medially than spinal motor neurons labeled following TA muscle injections. Representative images are from animals at the 42-h survival time (soleus) and the 44-h survival time (TA). Insets represent higher power images of the regions highlighted with asterisks. Scale bars in the main images represent 200 μm and those in the insets represent 50 μm. **(B)** The distribution of virally-labeled neurons along the rostral-caudal axis of the lumbosacral spinal cord from T13 to S2. The counts include all animals with first-order motor neuron labeling only following injection into either TA (*n* = 13; dashed line) or soleus (*n* = 11; solid line) muscles. The approximate locations within the spinal cord for the images provided in panel **(A)** are noted with # (TA) and + (soleus).

Rabies virus is a rhabdovirus with a marked neurotropism for the muscular form of the nicotinic acetylcholine receptor (Lentz et al., 1983). This neurotropism facilitates viral entry into cells at the neuromuscular junction. To assess the efficiency of transduction after injection, we evaluated the number of spinal motor neurons labeled prior to interneuron labeling (Table 1). For TA, this first-order labeling occurred at survival times ranging from 29–44 h. The number of motor neurons labeled was 4.7 ± 2.3 at 29 h, 14.0 ± 5.3 at 36 h, 18.0 ± 11.7 at 40 h, and 10.0 ± 7.2 at 44 h. The amount of labeling reached a maximum level between 36 and 40 h, with no increase at the longer survival time. First-order labeling following soleus muscle injections was observed at the 24-h survival time, with second order interneuron labeling observed as early as 42 h after viral injections. The amount of spinal motor neuron labeling for soleus was 16.0 ± 10.4 at 29 h, 23.0 ± 4.8 at 30 h, and 22.6 ± 5.7 at 36 h. For soleus, the amount of labeling reached a maximum by 30 h. The distribution of virally-labeled neurons along the rostral-caudal axis of the lumbosacral spinal cord details the location of these first-order motor neurons (Figure 2).

Following viral infection of motor neurons, rabies virus was transported trans-synaptically in the retrograde direction to interneurons (2nd order transport) and then, after another order of transneuronal transport, to cortical neurons in layer V (3rd order transport). Cortical layer V neurons are the cortical neurons with the most direct cortical projections to each muscle’s motor neurons. The earliest layer V labeling was observed at 60 h after TA injections and at 54 h following soleus injections. The number of layer V neurons labeled in those animals without 4th order labeling (i.e., labeling in layers II/III or VI) were counted (Table 2). For both muscles, layer V labeling continued to increase at longer survival times until the survival time at which labeling in cortical layers II/III and VI (4th order) was noted.

### 3.2. Anatomical nodes labeled via retrograde transneuronal transport

Spinal motor neuron labeling was assessed as the first relevant anatomical node and was used to establish the lower range of survival times necessary for transport (Figure 1). Labeling of these cells is associated with the retrograde transport of rabies virus from injected muscles via the neuromuscular junction to the motor neurons directly responsible for their control. First-order spinal motor neuron labeling was restricted to only one motor nuclei (representative images provided in Figure 2A) and observed in the appropriate lumbar segments (approximately L2-L5 for both TA and soleus) with an overlapping rostral to caudal distribution (Figure 2B). Labeling of these spinal motor neurons was first noted at 24 h for soleus and at 29 h for TA.

**FIGURE 3:**
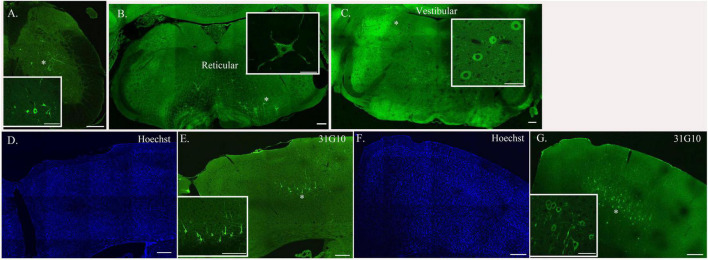
Neurons located at specific anatomical nodes are infected in a time-dependent manner following rabies virus injection. Representative immunofluorescence images following viral injections into either soleus or TA depict labeling in spinal interneurons [soleus, 54-h survival time **(A)**], brainstem neurons [TA, 69-h survival time **(B,C)**], and cortical neurons [TA, 93-h survival time **(D,E)**; soleus, 75-h survival time **(F,G)**]. Insets represent higher power images of the regions highlighted with asterisks. Scale bars in the main images represent 200 μm and those in the insets represent 50 μm.

The transition to second-order labeling was considered to be when the labeling of spinal cord interneurons and/or brainstem neurons was observed and indicates the retrograde transneuronal transport of rabies virus from motor neurons to neuronal cells with direct synaptic communication with these cells. This labeling was apparent at longer survival times (44–60 h for TA and 42–54 h for soleus) and included neurons in both the ipsilateral and contralateral spinal cord, as well as the area surrounding the central canal. Brainstem second-order premotor neurons were observed predominantly in the magnocellular reticular nucleus (Mc), pontine reticular nucleus (Pn), gigantocellular reticular nucleus (Gi), spinal vestibular nucleus (SpVe) and vestibular nucleus (Ve) following hindlimb muscle injections (Figure 3). There were no apparent differences in the extent of labeling at these sites following TA or soleus injections.

Third-order cortical labeling was subsequently observed at survival times of 58–84 h for TA and 54–67 h for soleus (Figure 3). The time course of this transport process was established for each hindlimb muscle and is further described below.

### 3.3. Cortical representations of hindlimb muscles

**FIGURE 4:**
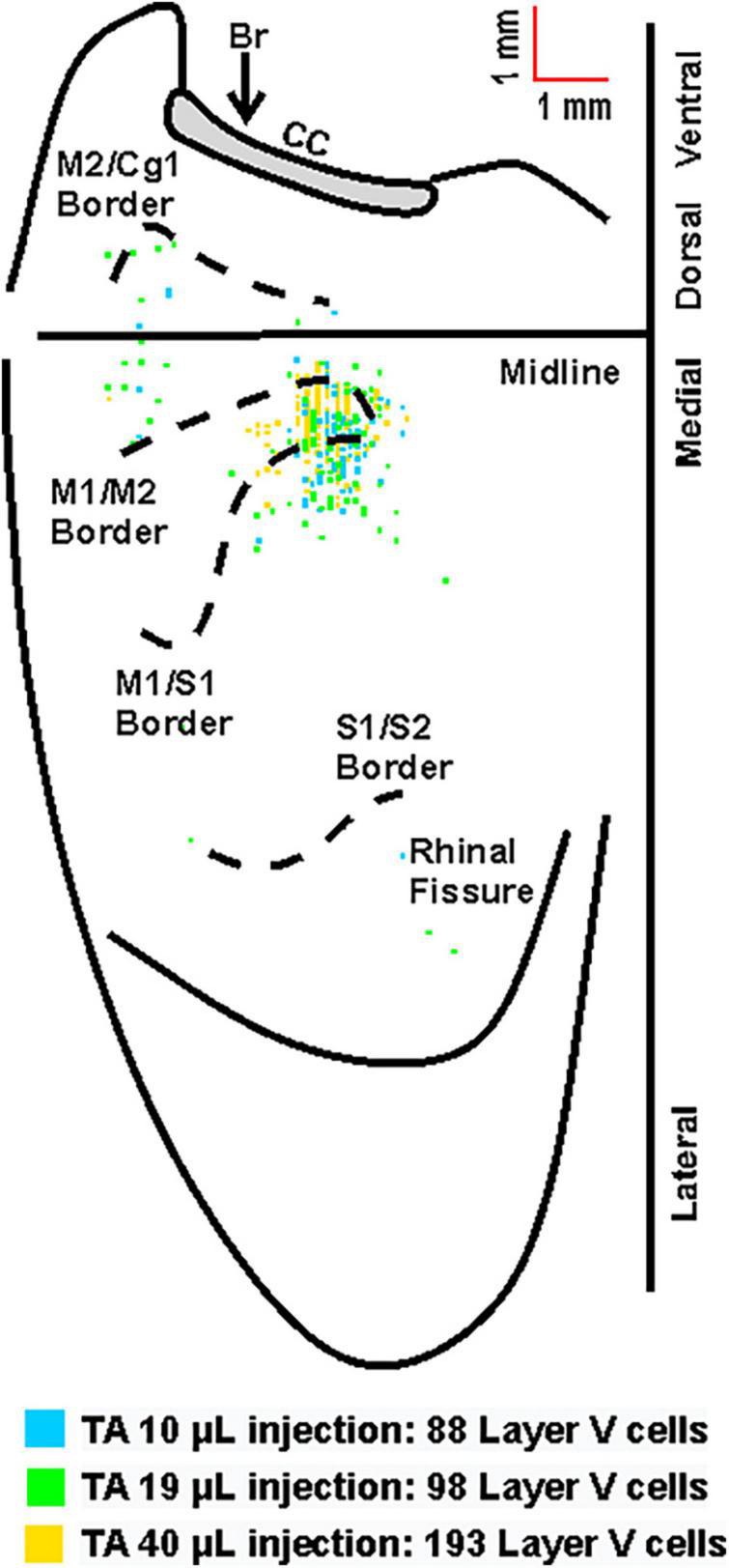
Overlay of the cortical representations of TA with varying injection volumes of rabies virus. Representative cortical maps from animals with third-order neuronal labeling (square = one cell) following rabies virus injections of 10 μL (blue), 19 μL (green), or 40 μL (yellow) show the origin of projections. This overlapping labeling was observed in the contralateral hemisphere after retrograde transneuronal transport of rabies virus from the right TA muscle. The medial wall of the contralateral hemisphere has been reflected upward and joined to the lateral surface at the midline. Cytoarchitectonic borders are delineated including the border between granular (S1) and agranular (M1) cortex in the region of the forelimb and hindlimb representations. Midline, midline of the hemisphere; M1, primary motor cortex; M2, secondary motor cortex (rostromedial motor field); S1, primary somatosensory cortex; S2, secondary somatosensory cortex; Cg1, cingulate cortex; CC, corpus callosum; Br, bregma.

The first infected cortical neurons observed were located in layer V, a major output layer of the cerebral cortex. The distribution of this labeling was consistent after varying volumes of rabies virus were injected (Figure 4). By increasing the survival time, an additional stage of retrograde transneuronal transport allowed for the infection of fourth-order neurons located in other cortical layers, specifically layers II/III and layer VI. As noted in prior studies in the rodent (Levinthal and Strick, 2012), the presence of rabies-positive neurons in layer V and the absence of rabies-positive cells in layers II/III is “an unequivocal marker that transport is restricted to those cortical neurons that are most directly connected to the muscle of interest.”

**FIGURE 5:**
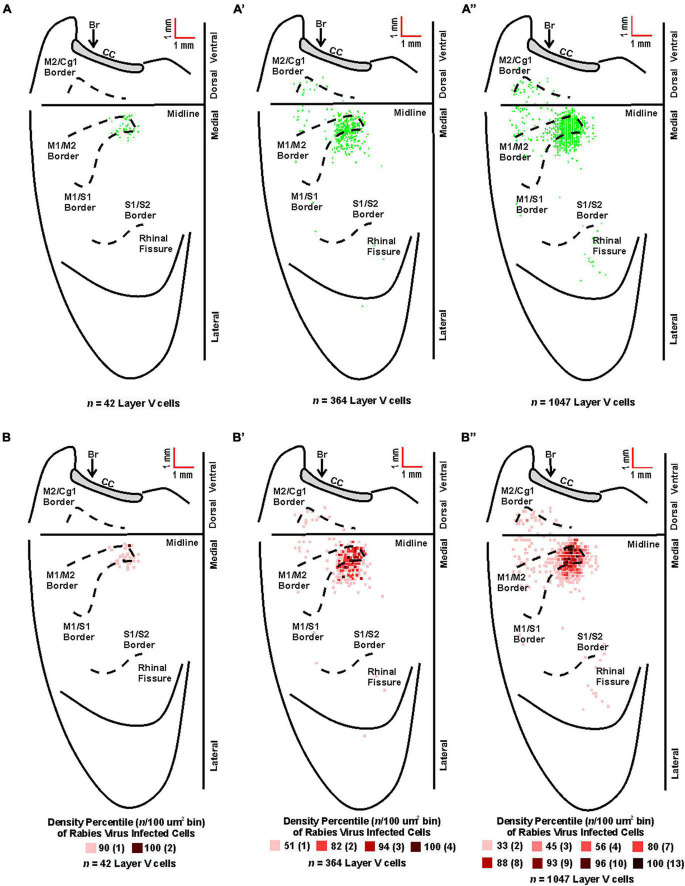
Origin of cortical projections to TA motor neurons. Top: Maps of third-order neurons (square = one cell) in layer V that were labeled in the contralateral hemisphere after retrograde transneuronal transport of rabies virus from the right TA muscle [**(A)** 63–69 h (*n* = 5); **(A’)** 75–84 h (*n* = 4); **(A”)** 84 h (*n* = 2)]. Each map is a composite of multiple experiments that were overlapped on the atlas template. Bottom: Density-based analysis of neuronal labeling in cortical layer V of the contralateral hemisphere after retrograde transneuronal transport of rabies virus from the right TA muscle [**(B)** 63–69 h (*n* = 5); **(B’)** 75–84 h (*n* = 4); **(B”)** 84 h (*n* = 2)]. Each square indicates the density of the infected neurons in bins (100 μm x 100 μm) throughout the cerebral cortex. Conventions and abbreviations as in Figure 4.

**FIGURE 6:**
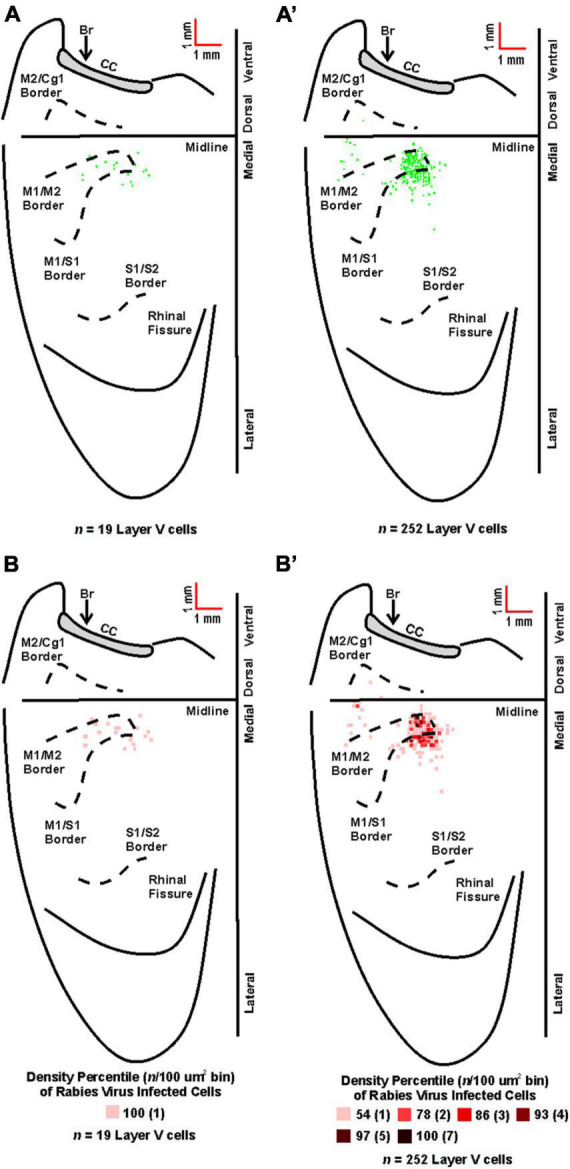
Origin of cortical projections to soleus motor neurons. Top: Maps of third-order neurons (square = one cell) in layer V that were labeled in the contralateral hemisphere after retrograde transneuronal transport of rabies virus from the right soleus muscle [**(A)** 60 h (*n* = 4); **(A’)** 67 h (*n* = 4)]. Each map is a composite of multiple experiments that were overlapped on the atlas template. Bottom: Density-based analysis of neuronal labeling in cortical layer V of the contralateral hemisphere after retrograde transneuronal transport of rabies virus from the right soleus muscle [**(B)** 60 h (*n* = 4); **(B’)** 67 h (*n* = 4)]. Each square indicates the density of the infected neurons in bins (100 μm × 100 μm) throughout the cerebral cortex. Conventions and abbreviations as in Figure 4.

For animals with labeling restricted to third-order, infected cortical neurons were located in layer V only and predominantly in the hemisphere contralateral to the injection site (100%, 81%, and 74% for TA at 63–69 h, 75–84 h, and 84 h, respectively; 95% and 86% for soleus at 60 h and 67 h, respectively). Following transport from TA, the cortical areas with infected neurons 63–69 h post-injection were the primary motor cortex (M1, 64%), the rostromedial motor area (M2, 12%), and the primary somatosensory cortex (S1, 24%). This distribution expanded to include additional cortical areas 75–84 h post-injection [M1 (45%), M2 (12%), S1 (41%), S2 (1%), and Cg1 (1%)] and 84 h post-injection [M1 (52%), M2 (16%), S1 (30%), S2 (1%), and Cg1 (1%)] (Figure 5). Similarly, following transport from soleus muscle, cortical layer V labeling was first observed at 60 h with peak labeling observed at 67 h. At 60 h, the cortical areas with infected neurons were M1 (47%), M2 (16%), and S1 (37%) with similar cortical areas labeled at 67 h post-injection [M1 (47%), M2 (9%), and S1 (44%)] (Figure 6). These motor and non-motor areas represent the most direct cortical influences on both TA and soleus.

**FIGURE 7:**
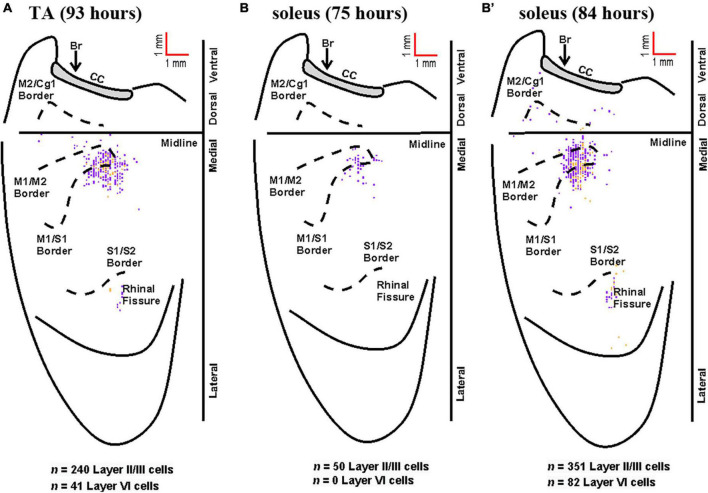
Representative cortical maps from animals with fourth-order labeling. Fourth-order cortical neurons are located most densely in the limb representations of M1, S1, and M2. **(A)** Following rabies virus injection into TA, infected neurons were observed in supragranular layers (layer IV and above, purple) and in layer VI (gold) at the 93-h survival time (map from a representative animal). **(B)** Following rabies virus injection into soleus, infected neurons were observed in supragranular layers (layer IV and above, purple) at the 75-h survival time (map from a representative animal). **(B’)** At the 84-h survival time, infected neurons were observed in supragranular layers (layer IV and above, purple) and in layer VI (gold) following rabies virus injection into soleus (map from a representative animal). All maps are from representative animals. Each square represents a single labeled neuron. Conventions and abbreviations as in Figure 4.

Rabies-positive neurons were observed in additional cortical layers when survival times were extended to allow for fourth-order labeling. These infected neurons in the supragranular cortical layers were localized predominantly in M1, M2, and S1 in regions comparable to those observed for third-order neurons in layer V at shorter survival times (Figure 7). Taken together, these observations indicate that M1, M2, and S1 are the origin of the most direct cortical control of these two hindlimb muscles and that this cortical control is mediated by interneurons located in the brainstem and spinal cord.

### 3.4. Cortical representations of fast- versus slow-twitch muscles

Fiber-type impacts a number of aspects of muscle physiology including energy usage, fatiguability, and recruitment order. To determine if differences in innervation patterns between fast- and slow-twitch muscles exist, we compared the anatomical nodes and neuronal populations labeled following rabies virus injection and found that fiber-types also impacts viral uptake. First, we observed spinal motor neuron labeling at shorter survival times after rabies virus injections into the slow-twitch soleus muscle when compared to the primarily fast-twitch TA muscle. Second, we describe differences in the time course of cortical labeling following soleus and TA injections that appear to reflect their differential muscle fiber composition.

**FIGURE 8:**
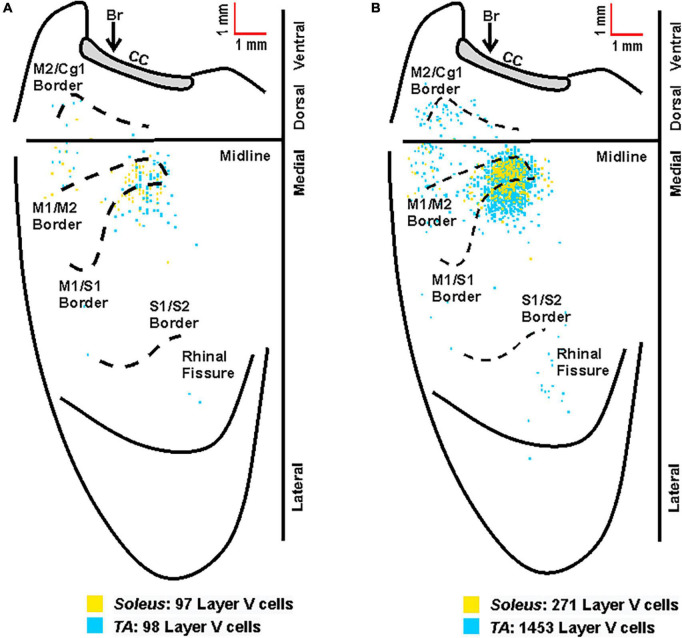
Overlay of the cortical representations of TA and soleus muscles. **(A)** Representative cortical maps from animals with third-order neuronal labeling following rabies virus injection into TA (blue) or soleus (yellow) show the origin of projections to each hindlimb muscle. **(B)** Composite cortical maps from animals with third-order only neuronal labeling used for the density analyses in Figures 5, 6 following RV injection into TA (*n* = 11; blue) or soleus (*n* = 8; yellow). The medial wall of the contralateral hemisphere has been reflected upward and joined to the lateral surface at the midline. Each square represents a single labeled neuron. Cortical projections originate from overlapping regions of M1, M2, and S1 for TA and soleus. Conventions and abbreviations as in Figure 4.

Overall, the anatomical location of the cortical neurons innervating the soleus and TA were consistent across the two muscles. In addition, the cortical representations in M1 showed significant overlap. However, the labeling after TA injections was somewhat more extensive than after soleus injections, in particular in S1. This is best observed when the cortical labeling associated with each muscle is overlaid (Figure 8).

### 3.5. Timing of retrograde transneuronal transport

**FIGURE 9:**
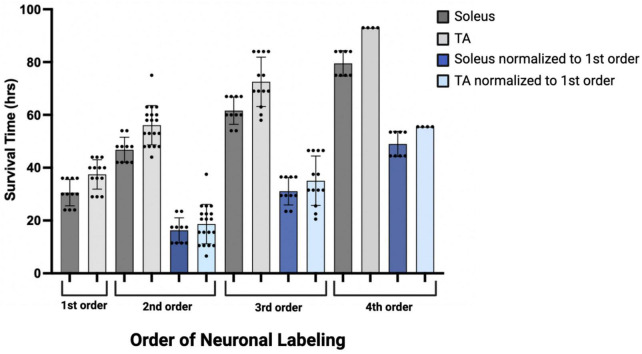
Descriptive graph of the time course of transport to locations throughout the motor system (orders of labeling as shown in Figure 1) following viral injection. Mean survival times (h) and standard deviations associated with first (*n* = 11, soleus; *n* = 13, TA), second (*n* = 10, soleus; *n* = 19, TA), third (*n* = 10, soleus; *n* = 13, TA), and fourth (*n* = 8, soleus; *n* = 4, TA) order labeling are provided (dark gray bars, soleus; light gray bars, TA). To delineate viral uptake at the neuromuscular junction from transneuronal transport, the timing of second, third, and fourth order labeling was normalized to the mean survival time associated with first order labeling (30.55 h for soleus and 37.46 h for TA; dark blue bars, soleus; light blue bars, TA).

Initial comparisons indicated that first-order labeling was observed at shorter survival times following viral injections for the soleus muscle compared to TA (soleus 30.55 ± 4.89 h, TA 37.46 ± 5.59 h; Figure 9, dark and light gray bars). Similar differences between TA and soleus were also observed at higher orders of transport—second order: soleus 46.80 ± 4.73 h, TA 56.10 ± 7.47 h; third order: soleus 61.60 ± 5.19 h, TA 72.54 ± 9.07 h; and fourth order: soleus 79.50 ± 4.81 h, TA 93.0 ± 0.00 h (Figure 9, dark and light gray bars). To determine if these differences were due to a differential rate of virus uptake from the predominantly (90% type II) fast-twitch TA versus the slow-twitch (50% type I) soleus, we normalized the higher order survival times by subtracting the mean survival time for first order labeling (37.46 h for TA; 30.55 h for soleus) from the survival times associated with second, third, and fourth order labeling. After normalization, the rates of retrograde transneuronal transport from TA or soleus were similar (Figure 9, dark and light blue bars). This descriptive analysis suggests that once the virus is transported retrogradely from the muscle to the motor neurons, further retrograde transneuronal transport occurs at the same rate regardless of the muscle of origin.

## 4. Discussion

In this study, we used retrograde transneuronal transport of rabies virus to identify and compare the cortical areas that control fast- and slow-twitch hindlimb muscles surrounding the ankle joint in the mouse and reported the density-based distribution of these cortical connections. Viral uptake from the slow-twitch soleus muscle injection and subsequent labeling of spinal motor neurons was observed at earlier survival times than viral uptake and spinal motor neuron labeling from the fast-twitch TA muscle injection. However, the time course for further retrograde transneuronal transport of rabies virus from spinal motor neurons to neurons in the cerebral cortex did not differ between the two muscles. Our data provides a foundation for future studies aimed at understanding disease-specific changes that occur in particular cellular populations within these neural networks and will specifically help to identify pathological alterations related to retrograde transneuronal transport.

Despite its central role in the study of disease, the mouse motor system has yet to be fully characterized, particularly with respect to hindlimb muscles that are less involved in the completion of sophisticated tasks (Esposito et al., 2014). In this study, the cortical areas associated with the control of hindlimb muscles were consistent with those noted in stimulation studies. In particular, the hindlimb was represented in the caudal portion of M1 and medial to S1, although overlap with the sensory cortex was also noted (Ayling et al., 2009; Tennant et al., 2011). Moreover, this hindlimb representation was predominantly located posterior to bregma and extended caudally as noted by others (Li and Waters, 1991; Pronichev and Lenkov, 1998). Most of the labeled layer V cortical neurons (3rd order) were located in the hindlimb representations of M1, M2, and S1 with additional labeling in regions (S2) surrounding the rhinal fissure. Also consistent with other studies (Wang et al., 2018; Steward et al., 2021), we did not observe significant overlap with forelimb regions of the sensorimotor cortex.

Notably, previous studies have identified a parallel circuit from the somatosensory cortex to lumbar motor neurons, suggesting that S1 plays a direct role in the control of locomotion (Karadimas et al., 2020). These authors identified a circuit that had the following chain of connections: layer V neurons in S1; propriospinal neurons in the cervical spinal cord (C4-5); locomotor pattern generator neurons (T13-L2), lumbar motor neurons. This circuit contains, at a minimum, four orders of transport and its cortical location should be within the cervical territory of corticospinal projections (Kamiyama et al., 2015). Our experiment focused on third order connections from the hindlimb muscles to the cortex (Figures 1, 5, 6). This labeling was restricted to the hindlimb representation of M1 and S1. We observed no apparent expansion of the labeled territory for TA and soleus projections into forelimb regions of M1 and S1 when labeling included 4th order neurons (compare Figures 5, 6 with Figure 7). Therefore, we are unlikely to have observed any labeled neurons belonging to the circuit reported by Karadimas and colleagues (Karadimas et al., 2020) in our experiments.

Third-order labeling of layer V cortical neurons following TA injection was first observed at 58 h and increased at longer survival times, reaching a peak at 84 h. When considered in the context of the staggered temporal infection reported previously for the TA motor neuron pool (Jovanovic et al., 2010), this graded increase in cortical labeling after TA muscle injections may reflect progressive virus uptake from gamma, small alpha, and then large alpha motor neuron populations of the TA muscle. Similar results were obtained following soleus muscle injections with early cortical labeling observed at 54 h with peak density of layer V neurons at 67 h. This pattern of labeling may correspond to progressive virus uptake from the gamma and then small alpha motor neurons of the soleus muscle. In addition, the layer V labeling could include both third- and fourth-order neurons as layer V corticospinal neurons in rodents can form synaptic connections with neighboring corticospinal neurons (Biane et al., 2015). However, for the cases defined as third-order, labeling was not observed in additional cortical layers which would be expected if fourth-order labeling was present. Further studies are needed to more fully explore the relationship between muscle fiber composition, virus uptake via different motor neuron subtypes, and timing of transport to cortical motor neurons. In particular, the reliability of the timing of viral uptake and transport to first-order spinal motor neurons will be explored to better understand the potential sample variance.

The overlap of the cortical representations of the two hindlimb muscles (Figure 8) has several important implications. First, we observed a clear separation between the hindlimb representation and areas involved in forelimb control (Tennant et al., 2011), consistent with previous findings in both rodents and non-human primates (Strick et al., 2021). In addition, our data indicate that cortical representations of single hindlimb muscles in mouse motor cortex are not unique, but rather exhibit considerable overlap and intermingling with other hindlimb muscles. This organization is in line with previous observations in the mouse forelimb (Ueno et al., 2018) as well as studies in the non-human primate using anatomical tracing to label single corticomotoneuronal (CM) cells (Rathelot and Strick, 2006) and stimulation to evoke muscle activity (Gould et al., 1986; Waters et al., 1990; Donoghue et al., 1992; Schieber, 2001; Graziano et al., 2002, 2005; Hudson et al., 2017). Second, our results indicate that hindlimb muscles with opposing physiological actions (i.e., ankle flexion for TA versus ankle extension for soleus) occupy the same cortical space in the mouse, consistent with findings in non-human primates (Hudson et al., 2013). Studies in non-human primates looking specifically at forelimb antagonist muscle representations have demonstrated that although these neurons occupy the same space in the cortex, they do not have overlapping branching patterns. In fact, Griffin et al. found that “…the multiple functions of a target muscle are represented by the activity of separate populations of CM cells” (Griffin et al., 2015). Together with our findings, these results support the conclusion that corticospinal neurons differ not only on the specific muscles and muscle fiber types to which they project but also on their functional utilization. Future studies using dual-tracer approaches in the same animal and/or physiology techniques are needed to determine whether individual corticospinal neurons map onto functionally distinct muscle types (e.g., flexors versus extensors) in the mouse motor cortex. Third, our results have important implications for pathological conditions like ALS and highlight that mechanisms beyond anatomical organization must be considered. In particular, differential neuronal loss within the same cortical space suggests that circuitry-, cellular-, and molecular-level factors may play a role in disease susceptibility. While muscle fiber type impacts multiple aspects of muscle physiology including energy usage, fatiguability, recruitment order, and rabies virus uptake (as observed in the present study), additional mechanisms (perhaps within the spinal cord and/or based on inherent cellular differences) may also have an impact. Our ongoing studies seek to evaluate the relationship between these factors and disease susceptibility.

TA motor neurons were first labeled 29 h following rabies virus injections into the muscle, and the number of motor neurons increased until 44 h post-injection when second order labeling was first noted in spinal interneurons. These results are consistent with those obtained using pseudorabies virus (PRV)-Bartha, a DNA virus. With PRV-Bartha, the TA motor neuron pool was first labeled 24–28 h following virus injection with increased labeling observed up to 72 h (Jovanovic et al., 2010). While the time course was similar, the number of motor neurons labeled with rabies virus was lower than that observed by others (Tissenbaum and Parry, 1991; Dekkers et al., 2004; Jovanovic et al., 2010). The counts reported in our study may be decreased as a result of the limited amount of virus injected into each muscle to minimize its spread and/or the limited uptake of virus at sites distal to the neuromuscular junction. For rabies virus injections into the soleus muscle, the majority of motor neurons were labeled by 30 h post-injection. Increasing the volume and number of injections into each muscle may be necessary to label a larger percentage of these cells. In addition, the relationship between muscle fiber type and factors such as receptor density at the neuromuscular junction, viral replication rate, and viral uptake must be considered. These will be explored in future studies such that further characterization of the size distribution and motor neuron subtypes can be accomplished. Interestingly, the number of cortical layer V neurons labeled following TA muscle injections was greater than the number of cortical layer V neurons labeled following soleus muscle injections (309.5 ± 251.84 versus 63.0 ± 29.45, Table 2). This suggests that more cortical input is dedicated to TA and is consistent with functional magnetic resonance imaging (fMRI) data demonstrating higher levels of activity in motor cortical areas for dorsiflexion compared with plantarflexion of the ankle (Trinastic et al., 2010). In addition, transcranial magnetic stimulation-based studies indicate that TA is task-specifically adapted to a broader range of movements compared to the soleus muscle which is less specific and more limited in its magnitude of modulation (Lauber et al., 2018). For both spinal cord and cortical cell counts, we acknowledge that our cell counts may be slightly increased due to the inclusion of partial cells. While stereological techniques would be optimal for quantitative cell counts, this concern was partially mitigated by using sections that were relatively thick in relation to the mean diameter of the cells being counted (Hedreen, 1998). In addition, rabies virus is an RNA virus that remains confined to the cytoplasm (Ugolini, 2010), resulting in a decreased density of reaction product in the nucleus. Therefore, we did not count cells without an apparent nucleus and lacking the initial portion of the apical dendrite as these cell profiles are more likely to be partial cells.

In this study, we have carefully adjusted survival times to optimize transport to the cerebral cortex and did not pursue a precise definition of the specific connectivity (Goulding et al., 2014) and microcircuit organization (Bikoff et al., 2016; Ziskind-Conhaim and Hochman, 2017) of spinal interneuron and brainstem populations. In addition, we noted variability in the order of labeling across animals as reported previously both *in vitro* and *in vivo* [reviewed in (Kelly and Strick, 2000)]. We also evaluated the time course of labeling in interneuron populations located both ipsilateral and contralateral to the injection site. As reported with PRV (Jovanovic et al., 2010), ipsilateral interneurons were first observed dorsal, ventral, and medial to the motor neuron pools of both hindlimb muscles, particularly in laminae V-VIII and near the central canal. This ipsilateral labeling occurred 44 h post-injection for TA with contralateral labeling in the spinal cord observed as early as 48 h. Following soleus muscle injections, this ipsilateral interneuron labeling was observed as early as 42 h, and contralateral interneuron labeling was consistently observed 48 h after soleus injections. We hypothesize that these interneuron populations include Renshaw cells and Ia inhibitory neurons as both are known to have direct synaptic projections to motor neurons (Renshaw, 1946) and future analyses will include characterization of these subpopulations. Furthermore, it is possible that sensory uptake of virus, specifically by group Ia afferent fibers innervating muscle spindles, impacted our results. This has been reported in other studies (Velandia-Romero et al., 2013; Zampieri et al., 2014) and would contribute to the progressive labeling observed with expected differences in time course as a function of the number of synaptic connections for alpha and gamma motor neurons. However, considering the direct muscle injections and shorter survival times used in this study, we expect these contributions to be minor.

Brainstem labeling was observed prior to cortical labeling following viral injections into both hindlimb muscles. These observations are consistent with the work of others indicating that brainstem regions have monosynaptic connections to spinal motor neurons and serve as a relay station between the spinal cord and brain (Esposito et al., 2014; Pivetta et al., 2014). It also suggests the mouse has “last-order interneurons” (Brownstone and Bui, 2010) with motor cortex projections to spinal cord and brainstem regions as described in the rat (Miller, 1987; Ba-M’Hamed et al., 1996; Gabbott et al., 2005). In other words, second-order neurons located in the spinal cord and brainstem mediate transport to third-order neurons located predominantly in M1, the region with the highest percentage of cortical cells labeled. Relatedly, the cortical labeling observed in layers II/III (i.e., fourth order labeling, Figure 7) was localized to regions associated with the labeling observed in layer V (i.e., third-order labeling, Figures 5, 6) at the shortest survival times. As noted in the rat motor cortex (Donoghue and Wise, 1982), these hindlimb motor representations overlap with the corresponding sensory representations in what has been termed an “overlap zone,” characterized by granule cells in layer IV and pyramidal cells in layer V (Tennant et al., 2011).

Overall, our results provide a foundation for ongoing work aimed at better understanding the impact of disease, particularly for conditions with pathological alterations of projections to and from the motor cortex (Commisso et al., 2018) or axonal transport deficits. The ability to evaluate reorganization in specific motor networks may also reveal aspects related to differential neuronal vulnerability and the impact of cell death at particular nodes in the network. For example, there is evidence that alpha and gamma motor neurons are differentially affected in diseases of the motor system (Lalancette-Hebert et al., 2016) and that the loss of specific spinal interneuron populations can lead to motor dysfunction (Salamatina et al., 2020). The ability to further investigate the associated circuitry in murine models may provide insights into these differences with potential avenues and targets for therapeutic interventions.

## Data availability statement

The original contributions presented in this study are included in the article/supplementary material, and further inquiries can be directed to the corresponding author.

## Ethics statement

This animal study was reviewed and approved by the Institutional Animal Care and Use Committee of the University of Pittsburgh.

## Author contributions

CK was responsible for the conception of the study as well as the experimental design and wrote the first draft of the manuscript. CK, LM, MB, TS, and AC contributed to the acquisition, analysis, and interpretation of the data including statistical analyses and figure preparation. All authors contributed to manuscript revision and have read and approved of the submitted version.
